# Supplementary material for: Reconstructing cell cycle and disease progression using deep learning
Source: Nat Commun. 2017 Sep 6;8:463. doi: 10.1038/s41467-017-00623-3 (PMC5587733; doi:10.1038/s41467-017-00623-3)
Supplement: Supplementary file 1 — Supplementary Information [file 41467_2017_623_MOESM1_ESM.pdf]

Description of Supplementary Files

File name: Supplementary Information
Description: Supplementary figures, supplementary notes and supplementary references.

File name: Supplementary Movie 1
Description: tSNE for visualizing the activations of the last layer of the neural network using a validation dataset with cell-cycling Jurkat cells. The reconstruction of cell cycle and the detection of a cluster of abnormal cells can be seen.

File name: Peer review file

Supplementary Note 1: Pseudotime-based reconstruction of cell cycle

To compare our deep learning results with the class of pseudotime algorithms [1, 2, 3, 4], we use Diffusion Pseudotime (DPT) [3] in the implementation of Scanpy [5]. DPT has recently been very favorably discussed by the authors of Monocle [6], one of the most established pseudotime algorithms [2], and is more robust than Wanderlust [1], the underlying algorithm of Ref. [4]. Using DPT, we infer the progression of Jurkat cells based on different sets of features:

- deep learning (this work),
- classical image features [7],
- marker intensity [4].

For this, we focus on predicting the DNA content of cells, which measures the progression of cell cycle during G1, S and G2 phase as in Fig. 3b. Deep learning outperforms classical image extraction techniques (Suppl. Fig. 1a versus b): Both in the tSNE and the pseudotime versus DNA content scatter, a high correlation is only visible in the case of deep learning (Pearson correlation of 0.56 versus 0.021). Note that the tSNE in Suppl. Fig. 1a shows the same data as Fig. 3b, but in a two-dimensional tSNE.

When using the marker expression directly as an input for the pseudotime reconstruction [4], the quality of the reconstruction depends on the number of informative features. Here, we consider the five-dimensional (Suppl. Fig. 1c) and the two-dimensional case (Suppl. Fig. 1d). In both cases, the first feature is the marker intensity measuring DNA content, and other features are non-specific classical image features. It is not astonishing that in the latter case (Suppl. Fig. 1d), pseudotime correlates very well with the DNA content. If, as done in Ref. [4], only the informative feature is used as an input, one obtains perfect agreement. It is important to note that this requires knowledge of the markers and measuring the known markers, both of which poses strong constraints on the problem of interest. Also, note that only the deep learning based features are not only able to reconstruct the process, but at the same time separate the cluster of abnormal cells (tSNE of Suppl. Fig. 1a, clearly marked in the 3-dimensional version Fig. 3b).

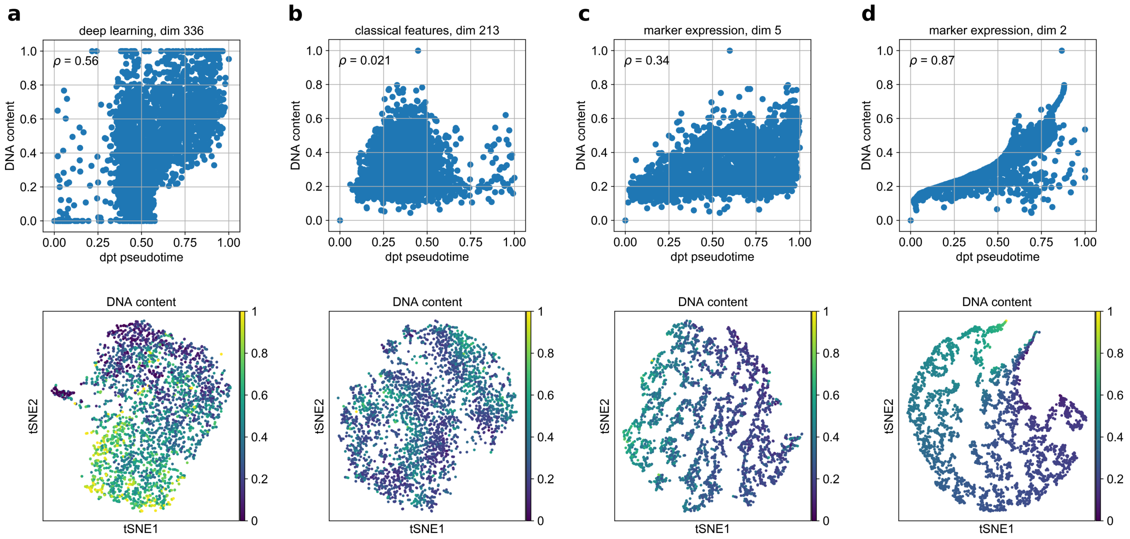

Supplementary Figure 1 | Comparison of pseudotime inference based on different feature sets. The first row shows scatter plots of DNA content versus pseudotime. The second row shows tSNE visualizations of the data represented in the respective features spaces. **a,** Deep learning (this work), **b,** classical features [7], **c,** marker intensity, analogous to Ref. [4], with a feature space of dimension 5, but here, only one dimension stores the marker that measures DNA content. **d,** Same as **c**, but for a feature space of dimension 2.

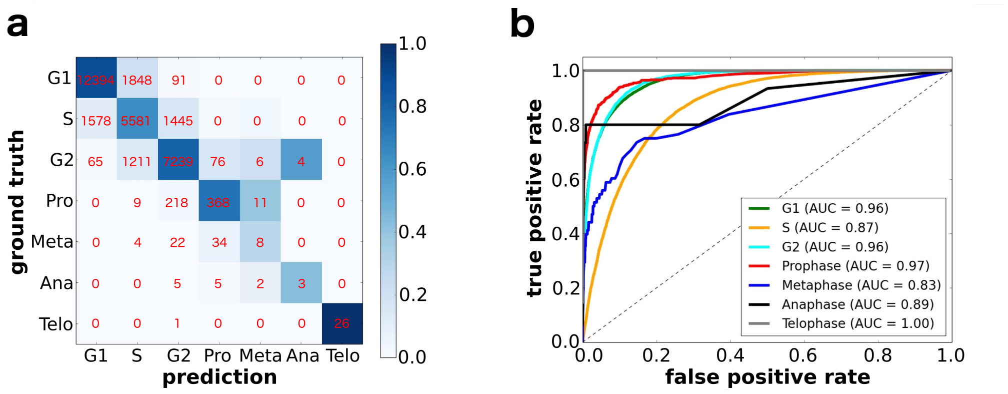

Supplementary Figure 2 | Performance of Deep learning for classification of seven classes. a, Confusion matrix. Red numbers denote absolute numbers of cells in each entry of the confusion matrix. Coloring of the matrix is obtained by normalizing absolute numbers to column sums, that is, diagonal elements correspond to precision. **b,** Class-specific Receiver Operating Characteristics.

Finally, note that the marker expression cannot only be used in pseudotime algorithms but also directly as an input for a regression based machine learning evalution. This has been studied for the cell-cycling Jurkat cells [7], who in addition to the classification discussed in the main text above, trained a regression boosting on the classical image features using the marker for DNA content as a continuous label. Not astonishingly, the results for this were much better (Pearson correlations of 0.786 to 0.894). The disadvantages of such an analysis and the advantages of our approach have already been discussed.

More visualizations and details on the analysis of this section are available from `https://github.com/theislab/scanpy_usage/tree/master/170529_images`.

Supplementary Note 2: Classification of all seven cell-cycle phases

We also evaluated the full seven-class problem in which the three interphase classes are considered individually. Here, we obtain an accuracy of 79.40%±0.77%. This number serves as an orientation for what deep learning could be able to achieve on this particularly hard classification problem — G1, S and G2 are extremely difficult to distinguish (see Fig. 2), even when using information from the fluorescence channels. The accuracy might therefore be affected by wrong labelling, it might be higher if all fluorescence channels were used as input for the neural network, and it might be slightly lower if "bleed through" enriched brightfield and darkfield images. If high classification accuracy is of importance, and one is not only interested in visualizing and interpreting the data, these questions have to be answered from case to case. Their answer depends in particular on how labels are generated and how many channels of the IFC are used.

Here, we confirm that the considerably lower accuracy as compared to the five-class problem results primarily from cells in the S phase being wrongly classified as either G1 or G2 (Suppl. Fig. 2a). This is also shown by the Receiver Operating Characteristic, which relates the true positive rate (sensitivity) with the false positive rate (fall-out) as the classification threshold changes (Suppl. Fig. 2b). Integrating the curve to obtain the standard performance metric "Area under the curve" (AUC). Even though the AUC for the S phase is still high with 0.87, it is the lowest among the majority classes (G1, S, G2), and therefore has a strong effect on the accuracy. Overall we find that all seven classes yield high values, greater than 0.85, and four of the seven classes, yield very high values, greater than 0.95.

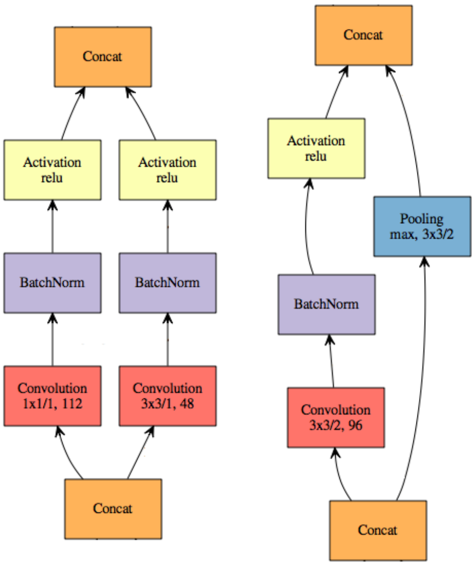

Supplementary Figure 3 | Dual-path modules. a, Normal dual-path module. **b,** Reduction dual-path module. The numbers beneath the convolution operations indicate the kernel sizes, stride and the number of filters.

Supplementary Note 3: Technical Notes

Preprocessing.

Our algorithmic workflow of cell cycle analysis with Deep Neural Networks begins with brightfield and darkfield images from the cells. In order to allow uniform training of our network on the whole dataset, we resize the images to 66 $\times$ 66 pixels by stretching the border pixels. We choose this method over individual image rescaling to avoid the destruction of possibly important size relation information between cells.

The data set used in this paper has been preprocessed using the IDEAS® software (Merck Millipore Inc.) to remove images of abnormal cells. In particular, we removed out of focus cells by gating for images with gradient RMS and removed debris by gating for circular cells with a large area.

Network architecture.

Supplemental Figure 3 shows normal and reduction dual-path modules, the basic elements of the deep learning architecture discussed in the main text. Kernel sizes, stride and number of filters are indicated in the figure.

The network architecture consists of 42 layers, which results in a total number of parameters of about 2 mio. It is build up starting with 3 dual-path reduction modules, followed by 10 normal dual-path modules, one pooling layer, one fully connected layer and the softmax layer. Each dual-path module consists in 3 layers: a convolution layer, a batch normalization layer and an activation layer. Although there is no "big" fundamental difference between dualpath and standard convolution modules, dual-path based networks tend to converge a little better in practice, since the gradient flow from pooling and convolution in the reduction module counteracts the vanishing gradient problem: not the entire gradient gets multiplied by approx 10^{-4} convolutional weight, pooling just lets it through.

In the first (input) layer, all IFC channels are combined in a linear operation by feeding them in the channel — which equals the color — dimension of the convolution input. This means the convolution uses kernels which convolve over all channels simultaneously. The number of 3$\times$3 kernel weights then is nine times the number of channels. Increasing the number of channels simply increases the "kernel depth" in the color dimension, and hence, is trivial.

We note that the specific choice of the architecture is a matter of experience and cannot be further justified. Readers might use these parameters to reproduce our results or produce similar results on similar problems. We also note that there is some freedom in the specific choice of the architecture, small modifications will not qualitatively alter the results.

Training details.

The network was trained for 100 epochs using stochastic gradient descent with standard parameters: 0.9 momentum, a fixed learning rate of 0.01 up to epoch 85 and of 0.001 afterwards as well as a slightly regularizing weight decay of 0.0005. Training took around 7 h and was stopped manually by inspecting convergence cross-entropy.

Implementation.

The DeepFlow architecture is available from `https://github.com/theislab/deepflow` and has been implemented using the MxNet framework [8] and run on a NVIDIA Titan X GPU. MxNet is lightweight, fast and memory efficient and available from `https://github.com/dmlc/mxnet`. Due to the fast progress in the development of deep learning software packages, in the meanwhile, we have also implemented and successfully tested our architecture using TensorFlow, which is available from `https://github.com/tensorflow/tensorflow`. The user might choose the software package according to personal preferences.

Nonlinear dimension reduction.

We use tSNE [9]. See Scanpy [5] for a package that provides several non-linear dimension reduction methods for visualizing single-cell data.

Bleed through.

The data acquired using the ImageStream was fully compensated using typical control images (see Ref. [10]) so the image tiffs would have minimal bleed through between channels. We could not detect even a slight indication of bleed through in the Jurkat cell data, neither upon inspection by eye, nor upon correlating the integrated intensity of each fluorescence channel with the integrated intensity of bright and darkfield channels, respectively. We then checked the existence of bleed through in the Cytometer used for data generation by switching off the light source of the brightfield channel, while keeping the fluorescence excitation on. We would then expect zero intensity in the brightfield images, but instead measured a slight intensity stemming from the fluorescence channels. This common technical aspect of IFC measurements merits an own investigation and will appear elsewhere. Here, our aim is to compare methodologies rather than to claim absolute levels of accuracy.

Supplementary References

[1] Bendall, S. C. *et al.* Single-cell trajectory detection uncovers progression and regulatory coordination in human B cell development. *Cell* **157**, 714–725 (2014).

[2] Trapnell, C. *et al.* The dynamics and regulators of cell fate decisions are revealed by pseudotemporal ordering of single cells. *Nature Biotechnology* **32**, 381–386 (2014).

[3] Haghverdi, L., Büttner, M., Wolf, F. A., Buettner, F. & Theis, F. J. Diffusion pseudotime robustly reconstructs branching cellular lineages. *Nature Methods* **13**, 845–848 (2016).

[4] Gut, G., Tadmor, M. D., Pe'er, D., Pelkmans, L. & Liberali, P. Trajectories of cell-cycle progression from fixed cell populations. *Nature Methods* **12**, 951–954 (2015).

[5] Wolf, F. A., Angerer, P. & Theis, F. J. Scanpy for large-scale single-cell analysis. *GitHub* (2017). URL https://github.com/theislab/scanpy.

[6] Qiu, X. *et al.* Reversed graph embedding resolves complex single-cell developmental trajectories. *bioRxiv* 110668 (2017).

[7] Blasi, T. *et al.* Label-free cell cycle analysis for high-throughput imaging flow cytometry. *Nature Communications* **7**, 10256 (2016).

[8] Chen, T. *et al.* Mxnet: A flexible and efficient machine learning library for heterogeneous distributed systems. In *In Neural Information Processing Systems, Workshop on Machine Learning Systems* (2016).

[9] van der Maaten, L. & Hinton, G. Visualizing data using t-sne. *Journal of Machine Learning Research* **9**, 2579–2605 (2008).

[10] Filby, A. *et al.* An imaging flow cytometric method for measuring cell division history and molecular symmetry during mitosis. *Cytometry Part A* **79A**, 496–506 (2011).
